# Cesarean section and increased body mass index in school children: two cohort studies from distinct socioeconomic background areas in Brazil

**DOI:** 10.1186/1475-2891-12-104

**Published:** 2013-07-25

**Authors:** Marcelo Zubaran Goldani, Marco Antonio Barbieri, Antônio Augusto Moura da Silva, Manoel Romeu Pereira Gutierrez, Heloisa Bettiol, Helena Ayako Sueno Goldani

**Affiliations:** 1 Department of Pediatrics, Faculty of Medicine, University of Rio Grande do Sul, Rua Ramiro Barcelos 2350, CEP 90035-903, Porto Alegre, RS, Brazil; 2 Department of Pediatrics, Faculty of Medicine of Ribeirao Preto, University of Sao Paulo, Avenida Bandeirantes 3900, Campus USP Monte Alegre, CEP 14049-900, Ribeirao Preto, SP, Brazil; 3 Department of Public Health, University of Maranhao, Rua Barão de Itapary 155, Centro, CEP 65020-070, Sao Luis, MA, Brazil

**Keywords:** Cesarean section, Vaginal delivery, Obesity, Body mass index, Cohort study

## Abstract

**Background:**

Recent studies have raised controversy regarding the association between cesarean section and later obesity in the offspring. The purpose of this study was to assess the association of cesarean section with increased body mass index (BMI) and obesity in school children from two Brazilian cities with distinct socioeconomic backgrounds.

**Methods:**

Two birth cohorts respectively born in 1994 in Ribeirao Preto, a wealthy city in Southeast, and in 1997/98 in Sao Luis, a less wealthy city in Northeast of Brasil, were evaluated. After birth, 2,846 pairs of mothers-newborns were evaluated in Ribeirao Preto and 2,542 in Sao Luis. In 2004/05, 790 children aged 10/11 years were randomly reassessed in Ribeirao Preto and 673 at 7/9 years in Sao Luis. Information on type of delivery, maternal and child characteristics, socioeconomic position and anthropometric measurements were collected after birth and at school age. Obesity was defined as BMI ≥ 95th percentile at school age.

**Results:**

Obesity rate was 13.0% in Ribeirao Preto and 2.1% in Sao Luis. Cesarean section was associated with obesity and remained significant after adjustment only in Ribeirao Preto [OR = 1.74 (95% CI: 1.04; 2.92)]. The association between cesarean section and BMI remained significant after adjustment for maternal schooling, maternal smoking during pregnancy, duration of breastfeeding, gender, birth weight and gestational age, type of school and, only in Sao Luis, pre-pregnancy maternal weight. In Ribeirao Preto children born by cesarean section had BMI 0.31 kg/m^2^ (95%CI: 0.11; 0.51) higher than those born by vaginal delivery. In Sao Luis BMI of children born by cesarean section was 0.28 kg/m^2^ higher (95%CI: 0.08; 0.49) than those born by vaginal delivery.

**Conclusion:**

A positive association between cesarean section and increased BMI z-score was demonstrated in areas with different socioeconomic status in a middle-income country.

## Background

Cesarean section (CS) is increasing markedly worldwide. In the United States, the CS rate has increased by 48% since 1996, reaching a level of 31.8% in 2007 [1]. In Brazil, CS has been associated with high socioeconomic status (SES) [2] and its rate has increased from around 15% in the 1970’s to 50% in 2011 [3], reaching 77% in some private clinics [4]. This trend is reflected in many parts of the world. In China, the most populous country in the world, CS rates are approaching 50% [5].

Overweight and obesity rates are also increasing across the world. In school children, they have been related to an increased risk for metabolic disorders in adulthood [6]. Many low- and middle-income countries present overweight and obesity rates among adults or children similar to those found in the US [7]. It appears that the rate of increase per year in the prevalence of overweight plus obesity is about 1% for adults and 0.5-0.7% for children in the US, Australia, United Kingdom and China [8]. However, higher BMI and overweight remain concentrated in higher socioeconomic groups in many but not all low- to middle-income countries [9,10]. In Brazil, considering the epidemiologic transition in the presence of rapid socioeconomic mobility, the obesity rate has increased modestly between 1974/75 and 1989, but has shown a sharp increase from 1989 to 2009 in all age ranges and socioeconomic strata [11].

The development of obesity is a complex process involving genetic susceptibility and environmental factors, both of which remain only partially understood. In such instances, the gut microbiota is being increasingly recognized as an important factor connecting genes, environment, and the immune system [12-14].

Recently we have shown in a Brazilian birth cohort study of 2,057 subjects that those who were born by CS had a significantly increased risk of obesity at 22–25 years of age [15]. One of the possible mechanisms proposed has been related to the type of birth, vaginal or cesarean delivery, which may have a role in this metabolic imprinting [16]. Recent data have revealed that the gut microbiota can be considered to be an environmental factor that modulates obesity and other metabolic diseases [17].

Further studies have raised controversy regarding the association between CS and obesity. In addition to our study [15], other cohort studies identified CS as a significant risk factor for obesity [18-21]. Nonetheless, this same risk of obesity in adulthood was not detected in other Brazilian birth cohort studies, but only among boys aged 4 years [22]. Recently, a meta-analysis indicated that CS was moderately associated with offspring overweight and obesity [23].

As the association of CS and obesity seems to be a fact for adults, we hypothesize that this may also be the case for schoolchildren, even from settings with distinct socioeconomic backgrounds and with different prevalences of CS and obesity. Thus, the objective of the present study was to provide a new contribution for this causal pathway, investigating the association between CS and BMI and obesity rates in two birth cohorts from different areas in Brazil with distinct socio economic status.

## Methods

This study was approved by the Research Ethics Committee of the University Hospital, Faculty of Medicine of Ribeirao Preto, University of Sao Paulo; and by the Research Ethics Committee of the University Hospital, Federal University of Maranhao, Brazil. All participants gave signed informed consent.

This is a study including two birth cohorts from two different Brazilian cities. These cohorts were set up to investigate perinatal health and to study the effect of early life factors on chronic non-communicable diseases in later life.

The first birth cohort started in 1994 in Ribeirao Preto (RP) in Sao Paulo state. RP is a city with a total population of 461,427 inhabitants, located in the most developed state in Southeastern Brazil and ranked 22nd in the Human Development Index, considering Brazilian cities [24].

The second birth cohort started in 1997 in Sao Luis (SL) in Maranhao state. SL is a city with a total population of 871,068 inhabitants and is one of the poorest state capitals in the Northeastern region, the least developed area of Brazil, ranked 1112nd in the Human Development Index [24].

In RP, 2,923 newborns were included corresponding to all live-births during a 4-month period in the 10 maternity hospitals in the city (from April to August 1994). After exclusion of multiple births (n = 65), 2,858 singletons remained. Missing information amounted to 5.8% and was due to early hospital discharge or refusal to participate [25].

In SL, a systematic stratified sample according to the number of births in each of the 10 maternity hospitals in the city was collected from March 1997 to February 1998. One out of 7 births was selected from each maternity hospital. A total of 2,541 births were initially selected and, after excluding multiple gestations (n = 50) and stillbirths (n = 48), a final sample of 2,443 live-births was included [25].

Anthropometric measurements were obtained from newborns in the two live-birth cohorts just after birth (birth weight and birth length) by trained staff. Information related to the gestational, delivery and post-delivery periods was obtained by means of a standardized questionnaire.

In 2004/2005, a randomized sub-sample from each of the original live-birth cohort samples was calculated for reassessment. The following categories were selected according to birth weight: <1,500 g [very low birth weight (VLBW)]; 1,500-2,499 g [low birth weight (LBW)]; 2,500-2,999 g [insufficient birth weight (IBW)]; 3,000-4,249 g [normal birth weight (NBW)]; and ≥4,250 g [high birth weight (HBW)]; the latter birth weight category corresponded to +2 SD from the mean. The lowest (< 2500 g) and highest (≥4,250 g) categories of birth weight were oversampled in order to increase the power of the study [25].

Children were searched at schools or at home in both cities. Parents or persons responsible for all VLBW, LBW and HBW and for a fraction of one out of three children in the remaining groups were invited to participate by telephone or mail. In RP, after exclusion of 48 deaths in the first year of life, 2,810 children were alive at one year of age; from these, 1,150 children were eligible for follow-up. The follow-up rate was 68.7%, 24 of them being VLBW, 145 LBW, 593 IBW and NBW, and 28 HBW, for a total of 790 children aged 10–11 years [25]. In SL, after exclusion of 65 deaths in the first year of life, 2,378 children were alive at one year of age; 926 of these children were eligible for follow-up. With a follow-up rate of 72.7%, 673 children from 7 to 9 years old were evaluated, five of them being VLBW, 76 LBW, 573 IBW and NBW, and 19 HBW. Details of the methodology and flow charts of the studies were published previously [25].

In both cities losses were due to the impossibility of locating the children, to migration, to the fact that the children were not enrolled in school, to refusal on the part of the parents or because two schools in SL did not allow the research team to contact the children.

The variables obtained at birth and late infancy in both live-birth cohorts considered in this study were: type of delivery (vaginal or cesarean section), maternal smoking during gestation (yes or no), maternal schooling in completed years (≤ 8, 9–11 and ≥ 12), newborn gender (male or female), preterm birth (<37 gestational weeks based on the last menstrual period), maternal weight before pregnancy informed by mothers (continuous variable collected only in SL). Due to small numbers in the VLBW and HBW groups, birth weight in grams was reclassified in three groups for analysis (≥ 3,000 g, 2,500 |- 3,000 g and < 2,500 g).

In 2004/2005, anthropometric measurements (weight and height) were obtained from the two subsamples. Body mass index (BMI) was calculated by dividing weight in Kg by height in m^2^. Obesity was defined as BMI ≥ 95th percentile according to gender and age in months [26]. Type of schooling was classified as public or private and exclusive breastfeeding duration was categorized into ≤ 30 days, > 30 days, and no breastfeeding.

### Statistical analysis

Samples of 771 individuals have an 80% power to detect a minimal difference of 4% in obesity rates (estimating the prevalence rate of obesity at 10%) between the exposed and the non-exposed group, with a 5% likelihood of type I error [27].

Due to the complex sampling design, children with low and high birth weights were over-represented in the samples. Therefore, the prevalence estimates and their standard errors were calculated taking into account the different probabilities of selection of each birth weight and preterm birth group. Stratification of the sample by birth weight was also taken into account [25].

In order to investigate the association between CS and obesity in school-aged children a multivariable logistic regression model was performed, using the covariables described above in the adjustment. Furthermore, a multiple linear regression model was performed in order to investigate the association between CS and BMI z-score, as a continuous variable, also using the same covariables for controlling of confounding. Because of differences in age and sex, BMI was standardized by them as recommended by the World Health Organization [28,29]. Missing data on covariates were excluded from the final models when missing values were less than 5%. In the case of maternal schooling a dummy variable for missing cases was included in the analysis.

## Results

In 2004/2005, at reassessment of the two birth cohorts studied, the obesity rate was 13.0% in RP, whereas in SL it was 2.1%. The CS rate was 54.5% in RP and 30.8% in SL. The prevalence of obesity in RP was significantly higher in children delivered by CS compared with those born by vaginal delivery (15.7% vs 9.8%, P = 0.022). In SL, despite obesity not being significantly associated with type of delivery (p = 0.091), the obesity rate in children born by CS was more than twofold higher than among those born by vaginal delivery (3.5% and 1.4% respectively). Obesity was also associated with higher level of maternal schooling in RP. On the other hand, there was no significant association among covariates and obesity in SL (Table 1).

**Table 1:** Prevalence rates of obesity according to covariables in Ribeirao Preto and Sao Luis, Brazil

Variables*	**Ribeirao Preto**			**Sao Luis**		
	**Total**	**Obese**		**Total**	**Obese**	
	**N%**	**n%**	**P**	**N%**	**n%**	**P**
Type of delivery			0.022			0.091
Vaginal	353 (45.5)	33 (9.8)		460 (69.2)	7 (1.4)	
Cesarean	437 (54.5)	71 (15.7)		213 (30.8)	8 (3.5)	
Maternal schooling at birth (years)			0.010			0.811
≤ 8	462 (58.3)	46 (10.0)		404 (60.6)	8 (1.9)	
9 – 11	170 (20.8)	34 (20.8)		255 (37.6)	7 (2.4)	
≥ 12	93 (11.9)	15 (12.7)		14 (1.8)	0 (0.0)	
Missing	65 (9.0)	9 (14.8)		0 (0.0)	0 (0.0)	
Maternal smoking during pregnancy			0.948			0.442
No	595 (80.6)	82 (13.3)		645 (96.0)	15 (2.2)	
Yes	160 (19.4)	19 (13.1)		28 (4.0)	0 (0.0)	
Birth weight (g)			0.159			0.157
≥ 3,000	447 (64.0)	70 (14.6)		458 (69.8)	7 (1.5)	
2,500|- 3,000	174 (26.1)	17 (9.9)		134 (23.6)	4 (3.0)	
< 2,500	169 (9.9)	17 (10.4)		81 (6.6)	4 (4.9)	
Preterm delivery			0.852			0.315
No	604 (87.9)	82 (13.1)		586 (88.3)	14 (2.2)	
Yes	186 (12.1)	22 (12.5)		87 (11.7)	1 (0.8)	
Newborn gender			0.152			0.056
Male	402 (50.8)	62 (14.8)		348 (51.7)	11 (3.1)	
Female	388 (49.2)	42 (11.1)		325 (48.3)	4 (1.0)	
Maternal schooling at follow up (years)			<0.001			0.851
≤ 8	455 (58.8)	39 (8.9)		317 (48.5)	6 (2.0)	
9 – 11	221 (27.7)	47 (21.2)		317 (48.7)	8 (2.2)	
≥ 12	106 (13.5)	17 (13.6)		21 (2.8)	1 (3.7)	
Duration of breastfeeding			0.242			0.372
>30 days	616 (80.8)	76 (12.2)		521 (79.4)	11 (1.8)	
No Breastfeeding	104 (12.0)	17 (18.6)		62 (9.4)	1 (2.0)	
≤ 30 days	60 (7.2)	10 (14.2)		71 (11.2)	3 (4.5)	
Type of school			0.405			0.555
Public	647 (83.0)	80 (12.5)		519 (78.2)	10 (1.9)	
Private	143 (17.0)	24 (15.4)		153 (21.8)	5 (2.7)	

*Missing values less than 5% were not shown in the table.

CS was significantly associated with obesity in RP but not in SL in the unadjusted analysis. After adjustment, CS remained associated with obesity in school-aged children in RP. Children born by CS presented a 74% higher prevalence rate of obesity at school age. There was a small change in the estimate after adjustment suggesting no significant confounding effect of the covariates included in this model. In SL there was no significant association between CS and obesity; however; the OR was markedly positive (OR = 1.66, 95% CI 0.44 to 6.33) (Table 2).

**Table 2:** Unadjusted and adjusted Odds Ratio for obesity according to type of delivery in Ribeirao Preto and Sao Luis, Brazil

	**Ribeirao Preto**		**Sao Luis**	
**Variable**	**Unadjusted OR**	**Adjusted OR**	**Unadjusted OR**	**Adjusted OR**
	**(95% CI)**	**(95% CI)**	**(95% CI)**	**(95% CI)**
Type of delivery				
Vaginal	1.00	1.00	1.00	1.00
Cesarean	1.72 (1.08 to 2.74)	1.74 (1.04 to 2.92)	2.44 (0.84 to 7.16)	1.66 (0.44 to 6.33)

*OR*, Odds ratio; *CI*, Confidence interval.

Models adjusted for maternal schooling (collected at delivery and follow up), maternal smoking during pregnancy, duration of breastfeeding, newborn gender, birth weight and gestational age, type of school and, only in Sao Luis, pre-pregnancy maternal weight.

In the unadjusted linear model performed aiming to investigate the association between CS and BMI as a continuous variable, children born by CS presented BMI z-scores about 0.31 to 0.43 higher than those born by vaginal delivery in the two cities in the unadjusted analysis. After adjustment the estimates reduced a little, to 0.31 in RP and 0.28 z-score in SL (Table 3).

**Table 3:** Simple and multiple linear regression models considering the association between type of delivery and BMI z-score in school-aged children in Ribeirao Preto and Sao Luis, Brazil

**Variable**	**Ribeirao Preto**		**Sao Luis**	
	**Unadjusted LC**	**Adjusted LC**	**Unadjusted LC**	**Adjusted LC**
	**(95% CI)**	**(95% CI)**	**(95% CI)**	**(95% CI)**
Type of delivery				
Vaginal	0.00	0.00	0.00	0.00
Cesarean	0.43 (0.24 to 0.62)	0.31 (0.11 to 0.51)	0.32 (0.15 to 0.51)	0.28 (0.08 to 0.49)

*LC*, Linear coefficient; *CI*, Confidence interval.

Models adjusted for maternal schooling (collected at delivery and follow-up), maternal smoking during pregnancy, duration of breastfeeding, newborn gender, birth weight and gestational age, type of school and, only in Sao Luis, pre-pregnancy maternal weight.

## Discussion

By using birth cohort data from two Brazilian cities with distinct socioeconomic backgrounds, this study showed that CS was associated with later risk of obesity in Ribeirao Preto, Southeastern Brazil, an area with high CS and obesity rates, but not in Sao Luis, Northeastern Brazil, where CS and obesity rates were lower. We were also able to demonstrate a significant association between CS and higher BMI z-score in school-aged children from these two Brazilian birth cohorts.

CS has been associated with adverse outcomes during the life course. From a very early adaptive period to long-term effects such as immune response leading to an increased risk for asthma and neuroendocrine diseases, changes influencing stress response during the life course have been associated with CS [30,31]. The results of some previously published studies regarding the association between CS and overweight/obesity were mixed. Ajslev *et al.* did not find an overall significant association between CS and overweight in children aged 7 years in a large Danish birth cohort. However, they observed that antibiotic use before 6 months of age was associated with increased risk of overweight among children of normal weight mothers delivered by CS and also described a tendency towards increased risk of overweight in boys delivered by CS [18]. We speculate that the small obesity rate (1.5%) and CS rate (12.4%) in that study could have not allowed the authors to detect a significant association between CS and obesity, despite the study’s large sample size. In our study, the age difference of the children in the two cities could be partially responsible for the higher prevalence of obesity in RP compared to SL; however, the prevalences are too different to be explained by the children’s age only.

In a newborn cohort study in the US, Rooney *et al.* reported that mode of delivery was associated with a 2.49 (95% CI: 1.10, 5.62) increased relative risk for obesity in childhood, but not in adolescence or early adulthood. The CS rate in that study was 7.8% and the obesity rate was 22.6% in childhood, 29.6% in adolescence and 14.1% in adulthood [19]. A case–control study from urban China also reported that CS was associated with an increased risk of obesity in preschool children [20]. In another study, Huh *et al.* evaluated 1,225 children at 3 years of age in Boston, USA, and described a 3-fold increase in obesity in those delivered by CS after adjusting for a number of confounders, in agreement with our findings for the RP cohort [21]. More recently, in the United Kingdom, Blustein *et al.* carried out a longitudinal study with 10,219 children and identified a significant association between CS and overweight in 11 year olds [32].

In a previous study, we also found an adjusted prevalence ratio of 1.58 (95% CI: 1.23, 2.02) for obesity among young adults delivered by CS in RP, which is in accordance with our childhood data from the same city presented here [15]. Barros *et al.*, using data from three different birth cohort studies from early childhood to young adulthood in Pelotas, Southern Brazil, did not obtain findings similar to ours in nearly all groups but boys aged four [22]; however, the prevalence of obesity (8.3%) and CS rate (27.3%) in the oldest cohort were smaller than those in our previous study in RP, where the prevalence of obesity was 13.3% and CS rate was 31.5%. They used several covariables such as maternal height, pre-pregnancy weight, maternal skin color, and birth order in the adjustment. Even though the effect of CS was no longer significant after adjustment, the prevalence ratio remained positive in all categories, suggesting that, despite the large sample, their study may not have had enough power to detect a possible small effect size of CS on obesity in Pelotas. On the other hand, as in RP there was a higher prevalence of obesity and CS, it is possible that this fact collaborated to a higher power of this study to detect the association.

Recently, Flemming *et al.* also presented discordant results to ours [33]. Similarly to Barros *et al.*, the authors did not find a significant association between CS and overweight/obesity. After adjustment the association between CS and overweight/obesity was no longer significant, but the odds ratio remained positive in all models. Low power to detect a significant association was not excluded by the authors. In this present study, CS was associated with obesity in the more developed city, RP, but not in the less developed one, SL. Differences in CS and obesity rates between RP and SL may explain the discrepant results. In RP, high CS and obesity rates may have allowed us to detect a significant association between CS and obesity, which persisted after adjustment. Conversely, SL presented smaller CS and obesity rates, which may have decreased the power to detect a significant association between CS and obesity. However, in the multiple linear analyses, we were able to show a significant positive association between CS and BMI z-score in the two birth cohorts.

We speculate again about the role of environmental issues as factors contributing to obesity since we had hypothesized that increasing rates of CS may play a role in the obesity epidemic worldwide [15]. The link might be the intestinal microbiota that seems to play a role in the interaction between host genotype and diet to modulate host physiology and metabolism. Recent data have shown that the intestinal microbiota can affect obesity [17] and that the newborn’s intestinal bacteria during the first days of life may be influenced by the type of delivery [34]. Moreover, intestinal colonization could have a long-lasting effect on general health [35].

Other mechanism could also be hypothesized. CS has also been associated with alteration of newborn hormonal milieu, characterized by, for example, lower concentrations of plasma leptin and ghrelin in human newborns and other orexigenic peptides in animal models, which are related to *post partum* appetite control and have been reported to be associated with an increased risk for later obesity [23,30,36].

The main strength of our study was to detect the significant association in two different locals with differing confounding structures, reinforcing the causal inference between CS and obesity. In addition, our findings were sustained by a larger number of covariates included in the models compared with our previous study [15] and a sample size able to detect significant association between CS and BMI z-score. The main limitations were the lack of data related to CS indication and maternal pre-pregnancy weight in RP. We also did not have information on whether CS occurred or not before labor was started.

In previous studies we showed that, in RP, a lower percentage of children who participated in the follow-up study was born to mothers with low schooling and who smoked ≥ 10 cigarettes per day during pregnancy in comparison to eligible children who did not participate, but there was no difference regarding offspring sex [25,37]. The proportion of children born by CS was higher among those who participated in the follow-up when compared to those who did not participate (55.3% and 49.5% respectively, p = 0.06). In SL, a lower proportion of children were born to mothers with high schooling and who gave birth to males among those who participated in the study in comparison to the eligible group who did not participate (25); there was no difference regarding smoking status between the two groups [37] and also for CS rates (31.6% and 34.5%, p = 0.194). Differences in birth weight and preterm birth were observed due to the complex sampling design of the study and were corrected by weighting. The differences in the participation rates, especially regarding CS rates in RP, could have increased the likelihood of selection bias and could have led to an overestimation of the association between CS and obesity in this city.

It is possible that the increasing CS rates observed worldwide are collaborating to the obesity epidemic. In Brazil, maybe the audit practice on the medical profession, legislated for by the government, to assess whether clinical standards of care are being met, could help to lower these rates, as well as the use of outside assessors would be helpful [2].

In conclusion, this study demonstrated a positive association between CS and increased BMI z-score in areas with different SES in a middle-income country. Our results pointed out that CS may be associated with obesity in areas with high CS and obesity rates. Possible mechanisms to explain this association are changes in gut microbiota or in the hormonal milieu induced by CS, which may increase the risk of obesity in later life.

## Competing interest

The authors declared that they have no competing interest.

## Authors’ contributions

MZG, MRPG, and HASG designed the research; HB and MAB provided essential materials (database); MZG, MRPG and AAMS analyzed the data; MZG and HASG wrote the draft of the paper; MZG, MAB, HB and AAMS revised the manuscript critically for intellectual content; MZG had primary responsibility for final content. All authors read and approved the final manuscript.

## References

[B1] Neu J Rushing J Cesarean versus vaginal delivery: long-term infant outcomes and the hygiene hypothesis Clin Perinatol 2011 38 321 331 10.1016/j.clp.2011.03.008 21645799 PMC3110651

[B2] Gomes UA Silva AA Bettiol H Barbieri MA Risk factors for the increasing cesarean section rate in Southeast Brazil: a comparison of two birth cohorts, 1978–1979 and 1994 Int J Epidemiol 1999 28 687 694 10.1093/ije/28.4.687 10480697

[B3] Victora CG Aquino EM do Carmo Leal M Monteiro CA Barros FC Szwarcwald CL Maternal and child health in Brazil: progress and challenges Lancet 2011 377 1863 1876 10.1016/S0140-6736(11)60138-4 21561656

[B4] Rebelo F da Rocha CM Cortes TR Dutra CL Kac G High cesarean prevalence in a national population-based study in Brazil: the role of private practice Acta Obstet Gynecol Scand 2010 89 903 908 10.3109/00016349.2010.484044 20583936

[B5] Lumbiganon P Laopaiboon M Gülmezoglu AM Souza JP Taneepanichskul S Ruyan P Attygalle DE Shrestha N Mori R Nguyen DH Hoang TB Rathavy T Chuyun K Cheang K Festin M Udomprasertgul V Germar MJ Yanqiu G Roy M Carroli G Ba-Thike K Filatova E Villar J World Health Organization Global Survey on Maternal and Perinatal Health Research Group Method of delivery and pregnancy outcomes in Asia: the WHO global survey on maternal and perinatal health 2007–08 Lancet 2010 375 490 499 10.1016/S0140-6736(09)61870-5 20071021

[B6] Sun SS Liang R Huang TT Daniels SR Arslanian S Liu K Grave GD Siervogel RM Childhood obesity predicts adult metabolic syndrome: the Fels Longitudinal Study J Pediatr 2008 152 191 200 10.1016/j.jpeds.2007.07.055 18206688 PMC3988700

[B7] Popkin BM Does global obesity represent a global public health challenge? Am J Clin Nutr 2011 93 232 233 10.3945/ajcn.110.008458 21159790 PMC3021421

[B8] Popkin BM Recent dynamics suggest selected countries catching up to US obesity Am J Clin Nutr 2010 91 284S 288S 10.3945/ajcn.2009.28473C 19906804 PMC2793114

[B9] Subramanian SV Perkins JM Özaltin E Davey Smith G Weight of nations: a socioeconomic analysis of women in low- to middle-income countries Am J Clin Nutr 2011 93 413 421 10.3945/ajcn.110.004820 21068343 PMC3021433

[B10] Mardones F Villarroel L Karzulovic L Barja S Arnaiz P Taibo M Mardones-Restat F Association of perinatal factors and obesity in 6- to 8-year-old Chilean children Int J Epidemiol 2008 37 902 910 18653517 10.1093/ije/dyn133

[B11] Instituto Brasileiro de Geografia e Estatística – IBGE (Brazilian Insitute of Geographics and Statistics) http://www.ibge.gov.br/home/estatistica/populacao/condicaodevida/pof/2008_2009_encaa/comentario.pdf (accessed 20 July 2012)

[B12] Musso G Gambino R Cassader M Obesity, diabetes, and gut microbiota: the hygiene hypothesis expanded? Diabetes Care 2010 33 2277 2284 10.2337/dc10-0556 20876708 PMC2945175

[B13] Reyes M Gahagan S Díaz E Blanco E Leiva L Lera L Burrows R Relationship of adiposity and insulin resistance mediated by inflammation in a group of overweight and obese Chilean adolescents Nutr J 2011 10 4 10.1186/1475-2891-10-4 21235793 PMC3032662

[B14] Matthews VL Wien M Sabaté J The risk of child and adolescent overweight is related to types of food consumed Nutr J 2011 10 71 10.1186/1475-2891-10-71 21702912 PMC3130644

[B15] Goldani HA Bettiol H Barbieri MA Silva AA Agranonik M Morais MB Goldani MZ Cesarean delivery is associated with an increased risk of obesity in adulthood in a Brazilian birth cohort study Am J Clin Nutr 2011 93 1344 1347 10.3945/ajcn.110.010033 21508088

[B16] Gronlund MM Lehtonen OP Eerola E Kero P Fecal microflora in healthy infants born by different methods of delivery: permanent changes in intestinal flora after cesarean delivery J Pediatr Gastroenterol Nutr 1999 28 19 25 10.1097/00005176-199901000-00007 9890463

[B17] Greiner T Bäckhed F Effects of the gut microbiota on obesity and glucose homeostasis Trends Endocrinol Metab 2011 22 117 123 10.1016/j.tem.2011.01.002 21353592

[B18] Ajslev TA Andersen CS Gamborg M Sørensen TI Jess T Childhood overweight after establishment of the gut microbiota: the role of delivery mode, pre-pregnancy weight and early administration of antibiotics Int J Obes 2011 35 522 529 10.1038/ijo.2011.27 21386800

[B19] Rooney BL Mathiason MA Schauberger CW Predictors of obesity in childhood, adolescence, and adulthood in a birth cohort Matern Child Health J 2011 15 1166 1175 10.1007/s10995-010-0689-1 20927643

[B20] Zhou L He G Zhang J Xie R Walker M Wen SW Risk factors of obesity in preschool children in an urban area in China Eur J Pediatr 2011 170 1401 1406 10.1007/s00431-011-1416-7 21365176

[B21] Huh SY Rifas-Shiman SL Zera CA Edwards JW Oken E Weiss ST Gillman MW Delivery by caesarean section and risk of obesity in preschool age children: a prospective cohort study Arch Dis Child 2012 97 610 616 10.1136/archdischild-2011-301141 22623615 PMC3784307

[B22] Barros FC Matijasevich A Hallal PC Horta BL Barros AJ Menezes AB Santos IS Gigante DP Victora CG Cesarean section and risk of obesity in childhood, adolescence, and early adulthood: evidence from 3 Brazilian birth cohorts Am J Clin Nutr 2012 95 465 470 10.3945/ajcn.111.026401 22237058 PMC3260073

[B23] Li HT Zhou YB Liu JM The impact of cesarean section on offspring overweight and obesity: a systematic review and meta-analysis Int J Obes (Lond) 2013 37 893 899 10.1038/ijo.2012.195 23207407

[B24] Programa das Nações Unidas para o Desenvolvimento. (United Nations Development Programme) http://www.pnud.org.br/atlas/ranking/IDH_Municipios_Brasil_2000.aspx?indiceAccordion=1&li=li_Ranking2003 (accessed 15 March 2012)

[B25] Silva AA Barbieri MA Cardoso VC Batista RF Simões VM Vianna EO Gutierrez MR Figueiredo ML Silva NA Pereira TS Rodriguez JD Loureiro SR Ribeiro VS Bettiol H Prevalence of non-communicable diseases in Brazilian children: follow-up at school age of two Brazilian birth cohorts of the 1990’s BMC Public Health 2011 11 486 10.1186/1471-2458-11-486 21693042 PMC3141455

[B26] Cole T Bellizi M Flegal K Dietz W Establishing a standard definition for child over-weight and obesity worldwide: international survey BMJ 2000 320 1240 1243 10.1136/bmj.320.7244.1240 10797032 PMC27365

[B27] Cardoso VC Simões VM Barbieri MA Silva AA Bettiol H Alves MT Goldani MZ Profile of three Brazilian birth cohort studies in Ribeirao Preto, SP and Sao Luis, MA Braz J Med Biol Res 2007 40 1165 1176 10.1590/S0100-879X2006005000148 17713669

[B28] WHO Expert Commitee Physical status: the use and interpretation of anthropometry WHO Technical Reports Series; 854 1995 Geneva: World Health Organization 8594834

[B29] De Onis M Lobstein T Defining obesity risk status in the general childhood population: Which cut-offs should we use? Int J Pediatr Obes 2010 5 458 460 10.3109/17477161003615583 20233144

[B30] Hyde MJ Mostyn A Modi N Kemp PR The health implications of birth by Caesarean section Biol Rev Camb Philos Soc 2012 87 229 243 10.1111/j.1469-185X.2011.00195.x 21815988

[B31] Tollanes MC Moster D Daltveit AK Irgens LM Cesarean section and risk of severe childhood asthma: a population-based cohort study J Pediatr 2008 153 112 116 10.1016/j.jpeds.2008.01.029 18571547

[B32] Blustein J Attina T Lui M Ryan AM Cox LM Blaser MJ Trasande L Association of cesarean section delivery with child adiposity from 6 weeks to 15 years Int J Obes 2013 37 900 906 10.1038/ijo.2013.49 PMC5007946 23670220

[B33] Flemming K Woolcott CG Allen AC Veugelers PJ Kuhle S The association between caesarean section and childhood obesity revisited: a cohort study Arch Dis Child 2013 98 526 532 10.1136/archdischild-2012-303459 23680850

[B34] Biasucci G Rubini M Riboni S Morelli L Bessi E Retetangos C Mode of delivery affects the bacterial community in the newborn gut Early Hum Dev 2010 86 Suppl 1 13 15 20133091 10.1016/j.earlhumdev.2010.01.004

[B35] Kalliomaki M Collado MC Salminen S Isolauri E Early differences in fecal microbiota composition in children may predict overweight Am J Clin Nutr 2008 87 534 538 18326589 10.1093/ajcn/87.3.534

[B36] Hyde MJ Griffin JL Herrera E Byrne CD Clarke L Kemp PR Delivery by Caesarean section, rather than vaginal delivery, promotes hepatic steatosis in piglets Clin Sci 2010 118 47 59 19445654 10.1042/CS20090169

[B37] Fabbri CE Barbieri MA Silva AAM Gutierrez MR Bettiol H Speciali JG Rona RJ Maternal smoking during pregnancy and primary headache in school-aged children: a cohort study Cephalalgia 2012 32 317 327 10.1177/0333102411436261 22290555

